# Influence of different regimens of volumetric therapy on perioperative intestinal flora in the surgical patients with pancreas tumor, a randomized controlled trial study

**DOI:** 10.1186/s12871-022-01693-7

**Published:** 2022-05-25

**Authors:** Xiaojian Lu, Ying Wang, Yan Luo, Buwei Yu

**Affiliations:** Department of Anesthesiology, Ruijin Hospital Affiliated to Shanghai Jiao Tong University, Shanghai, China

**Keywords:** Perioperative period, Volumetric therapy, Intestinal flora

## Abstract

**Background:**

It is not clear whether the perioperative intestinal microenvironment of patients undergoing pancreatic tumor surgery is affected by intraoperative fluid therapy.

**Method:**

Fifty-eight patients who underwent a confined excision of pancreas mass at this center were enrolled. The patients were grouped according to the random number table in these two groups: the liberal fluid infusion (LFI) group and the goal-directed fluid therapy (GDFT) group. Perioperative anesthesia management was carried out by the same team of anesthesiologists according to a preset anesthetic protocol. Fecal samples were collected twice: within 2 days before the surgery and at 6 to 8 days postoperatively. The collected fecal samples were sequenced through microbial diversity high-throughput 16 s-rDNA; and the differential changes of intestinal flora were analyzed.

**Results:**

Main components of flora in the sample were significantly different between LFI and GDFT groups. As shown by the difference in species, in GDFT group, more constituent bacteria participated in the metabolism inside human body and the restoration of coagulation function, including: *prevotella*, *roseburia*, *lachnospiracea*, *dialister* and *clostridium* (*P* < 0.05); in LFI group, more constituent bacteria were opportunistic pathogenic bacteria, including: *enterococcus*, *pseudomonas aeruginosa*, and *acinetobacter baumannii* (*P* < 0.05).

**Conclusion:**

For surgical patients with pancreas tumor, there are significant differences of intestinal flora in diversity between GDFT and LFI. GDFT seems to play a more important role in protection and restoration of intestinal flora.

**Clinical trial registration:**

ChiCTR2000035187.

## Background

Intestinal flora is an important component of human body [1]. The stable diversity and quantity of intestinal flora are related to preoperative diseases in patients, including hypertension [2–6], coronary heart disease [7] and endocrine disorder. Factors, such as surgical stress [8, 9], systemic inflammatory reactions [10] and insufficient perfusion of internal organs caused by the perioperative hemodynamic change, ischemia and anoxia [11] may occur in intestinal mucosa and influence the intestinal flora in patients. In recent years, precision anesthesia more and more becomes basic requirements in clinical work. Reducing the damage of perioperative intestinal flora and promoting the restoration of postoperative intestinal flora might improve patient outcome after surgery [12].

The anesthesiologists are faced daily with several principal and practical problems when arranging perioperative fluid handling. Results of studies on fluid therapy [13–15] will have an impact on everyday practice only if clinicians are able to accept one or more alternative regimens as being superior. Therefore, a hypothesis was made in this study that the effect for protecting the diversity of perioperative intestinal flora and promoting its restoration varied with the mode of intraoperative fluid infusion in the surgical patients with pancreas tumor.

## Methods

### Ethics

This randomized controlled study was approved by the Ethics of Ruijin Hospital and registered under Chinese Clinical Trial Registry in 02/08/2020. The registration number is ChiCTR2000035187. All subjects provided written informed consent before surgery.

### Patients

Patients undergoing pancreaticoduodenectomy were selected. Within 2 days before the surgery, fecal samples were retained for the first time. These patients were randomly allocated into LFI group (group F) and GDFT group (group G) to adopt different modes of intraoperative fluid infusion. Within 6–8 days after the surgery, fecal samples were collected for the second time. In both group F and group G, fecal samples collected before surgery are named Pre-F group and Pre-G group, and similarly fecal samples collected after surgery are divided into subgropus, Post-F and Post-G group. Between two groups, preoperative and postoperative intestinal flora was compared respectively.

There were 58 enrolled subjects in this study (see CONSORT Flow Diagram). These patients were randomly allocated into LFI group (group F, 28 patients) and GDFT group (group G, 30 patients). Between two groups, general conditions of patients were not significantly different (Table 1).

**Table 1:** General conditions of patients

	Group F	Group G
**Age (year)**	62.29 ± 6.0	62.06 ± 7.5
**Gender (male/female)**	(13/15)	(15/16)
**Height (cm)**	168.0 ± 8.8	165.6 ± 7.8
**Weight (kg)**	65.1 ± 11.3	61.3 ± 9.6
**BMI (kg/m**^**2**^**)**	22.9 ± 3.2	22.3 ± 2.9
**Smoking history (smoking/total patients)**	12/28	12/31
**Drinking history (drinking/total patients)**	11/28	9/31
**Stage of tumor (AJCC)**		
**IIa**	8	10
**IIb**	8	9
**III**	5	7
**IV**	7	5

Between two groups, general conditions of patients and stage of tumor were not significantly different. BMI: Body Mass Index; AJCC: American Association of Cancer.

### Anesthesia procedures

After the induction of anesthesia but before the starting of surgery, the following drugs were dripped intravenously at uniform velocity: cefuroxime sodium 2.25 g + sodium chloride injection 250 mL; 500 mg metronidazole 100 mL.

In Group F, anesthetic depth and body temperature was monitored; When haemoglobin (Hb) was < 80 g/L, blood transfusion was given. Intraoperative fluid infusion was regulated by the fixed anesthesiologist.

In Group G, Parameters such as stroke volume variation (SVV) and cardiac index (CI) were monitored through FloTrac/EV1000™ device. According to the monitoring results, fluid infusion was given to maintain SVV at ≤13%. When SVV was > 13%, succinylated gelatin injection or blood products was rapidly infused intravenously until SVV was ≤13%; when MAP was < 60 mmHg, ephedrine was given; when CI was < 2.5, dopamine (2 ~ 3 μgkg^− 1^ min^− 1^) was infused intravenously via micropump. During the surgery, blood gas was monitored. When Hb was < 80 gL^− 1^, blood transfusion was given.

After the surgery, cefoperazone and sulbactam sodium for injection, 1500 mg bid was given as antibiotics in all patients. Same intravenous nutrients were daily given and adjusted as needed by the pancreas surgeon. After assessment, fluid diet was given after the surgery. Somatostatin 0.1 mg was daily injected intravenously until 8 days after the surgery and pancreatin enteric-coated capsules (Creon) was given daily.

### Sample analysis

In both groups, food and drinking were forbidden conventionally for 8 hours before the surgery; laxatives were not given within 2 days before the surgery. After patient urinates, fecal samples were collected and rapidly put into − 80 °C refrigerator at keeping ready for further analysis. By referring to the study protocol of Muyzer et al. [16], 1 g feces were taken from each sample; after the centrifuge, DNA extraction was made. At first, full-length amplification was made on 16S rRNA of total DNA in each sample through the primer of (5′-3′): CCTACGGGRSGCAGCAG (341F) and (5′-3′): GGACTACVVGGGTATCTAATC (806R) [17]. Specific primer setting was completed. In the conserved region at 5′ terminal and 3′ terminal of 16S rRNA gene, almost all of 16S rDNA gene were amplified. 16S specific primers were designed to amplify specific regions, and 425 bp amplified fragments were obtained. Illumina platform was used to obtain the paired end data, and a long sequence was obtained by splicing, so as to conduct 16S analysis.

### Statistical methods

In this study, a hypothesis was made that the effect for changing postoperative intestinal flora was different between intraoperative LFI and intraoperative GDFT in the pancreas tumor patients of confined surgery. Sample size was estimated through GPower 3.1 software and by t test. Effect size was set as 0.8; α error was set as 0.05; statistical power (1-β) was set as 0.8 [18]. The patients were randomly allocated into two groups in equal number. As calculated through above values, 26 patients were allocated into each group; sample size was preliminarily set as 52 cases.

The statistical analysis of our data was conducted in IBM SPSS 24. In both groups, demographic data of patients were analyzed statistically through mean value ± standard deviation and constituent ratio; t test on independent sample was made for the clinical data. All statistical tests were two-sided test: size of test (α) = 0.05. It was considered that the difference was statistically significant when *P* < 0.05. Data of intestinal flora were expressed with mean value. Statistical analysis was made through R programming language software. Through usearch software, cluster analysis was made on the qualified data. According to the descending order of abundance, RNA fragment of sample was clustered to obtain operational taxonomic unit (OTU). Each OTU represented one species. The reading value of each sample matched to OTU was summarized. From each OTU, one read was separately selected as the sequence representing this OTU; the species indicated by each OTU was classified to obtain the table of species abundance; according to the table of species abundance, subsequent analysis and calculation were made to obtain alpha diversity, beta diversity, including Weighted Unifrac diversity analysis and Adonis analysis. We use linear discriminant analysis (LDA) and LDA effective size analysis (LEfSe) to illustrate species difference at the level of genus of intestinal flora in each sample.

## Results

Before surgery, blood biochemical indices were not significantly different between two groups (Table 2). After the surgery, the following study parameters were not statistically different: VAS score at 1 and 3 days after the surgery (VAS 1 and VAS 3); highest body temperature at Day 1, 2, 3 and 7 after the surgery (T1, T2, T3 and T7); passing flatus time; and hospitalization duration. During the surgery, total fluid infusion volume and urine volume were statistically different between two groups (Table 3).

**Table 2:** Baseline biochemical indices of patients prior to surgery

	Group F	Group G
**White blood cell (×10**^**9**^ **L**^**− 1**^**)**	6.12 ± 1.33	5.14 ± 1.44^a^
**Red blood cell (×10**^**12**^ **L**^**− 1**^**)**	4.32 ± 0.47	4.25 ± 0.33^a^
**Haemoglobin (gL**^**−1**^**)**	136.9 ± 12.5	129.9 ± 13.7^a^
**Platelet (×10**^**9**^ **L**^**−1**^**)**	202.1 ± 57.4	208.9 ± 66.2^a^
**Total bilirubin (umolL**^**−1**^**)**	36.5 ± 42.0	41.6 ± 61.4^a^
**Total protein (gL**^**−1**^**)**	69.1 ± 6.1	67.5 ± 5.4^a^
**Albumin (gL**^**−1**^**)**	40.7 ± 3.6	39.8 ± 4.1^a^
**Creatinine (umolL**^**−1**^**)**	71.0 ± 15.7	68.2 ± 13.6^a^

^a^Compared with Group F, *P *> 0.05

**Table 3:** Comparison on the conditions of patients during and after the surgery

	Group F	Group G
**Surgical duration (minute)**	333.8 ± 66.2	320.7 ± 85.9
**Intraoperative total fluid infusion volume (mL)**	3808.9 ± 431.6	2514.5 ± 581.9^**a**^
**Intraoperative blood transfusion volume (mL)**	610.7 ± 585.2	625.8 ± 512.5
**Intraoperative blood loss (mL)**	423.2 ± 214.9	477.4 ± 262.9
**Intraoperative urine volume (mL)**	1344.6 ± 668.5	985.5 ± 567.2^**b**^
**Intraoperative dose of dopamine (mg)**	0	0.7 ± 1.97^**c**^
**Postoperative white blood cell (**^**×**^**10**^**9**^**L**^**− 1**^**)**	13.14 ± 4.3	12.84 ± 4.8
**Postoperative red blood cell (**^**×**^**10**^**12**^**L**^**− 1**^**)**	4.00 ± 0.5	4.01 ± 0.48
**Postoperative haemoglobin (gL**^**− 1**^**)**	125.6 ± 17.3	122.7 ± 15.4
**Postoperative platelet (**^**×**^**10**^**9**^**L**^**−1**^**)**	164.4 ± 41.8	180.5 ± 55.0
**Postoperative total bilirubin (umolL**^**−1**^**)**	41.5 ± 46.8	45.1 ± 39.0
**Postoperative total protein (gL**^**−1**^**)**	58.6 ± 6.9	58.5 ± 5.3
**Postoperative albumin (gL**^**−1**^**)**	34.6 ± 5.3	35.3 ± 4.7
**Postoperative creatinine (umolL**^**−1**^**)**	66.3 ± 15.4	67.7 ± 12.8
**VAS 1**	4.9 ± 0.7	5.0 ± 0.75
**VAS 3**	1.4 ± 0.5	1.7 ± 0.6
**T1**	37.6 ± 0.3	37.3 ± 0.4
**T2**	37.6 ± 0.4	37.5 ± 0.4
**T3**	37.7 ± 0.5	37.6 ± 0.5
**T7**	37.3 ± 0.4	37.2 ± 0.4
**Passing flatus time (day)**	3.8 ± 1.5	3.7 ± 1.3
**Postoperative application of enteric nutrients (day)**	6.0 ± 0.8	5.9 ± 0.9
**Postoperative hospitalization duration (day)**	17.5 ± 5.3	16.2 ± 3.9

^**a**^ Compared with Group F, *P* = 0.01

^b^ Compared with Group F, *P* = 0.03

^c^ Compared with Group F, *P* = 0.06

### OTU analysis

The species indicated by each OTU was classified to obtain the table of species abundance, which was used for subsequent analysis.

**Fig. 1:**
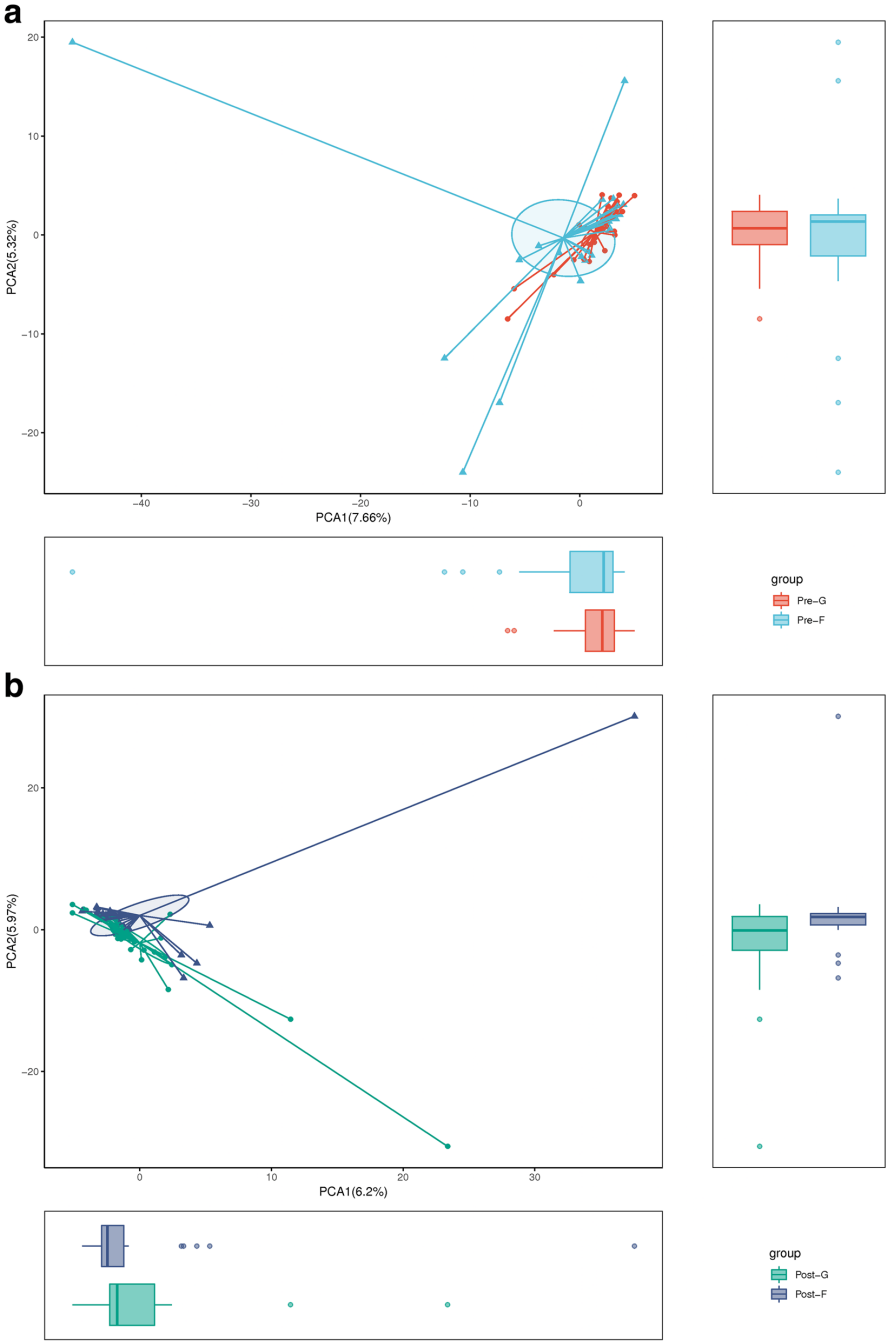
**a** Preoperative comparison of OTU level between the two groups. PCA1 is the significance of the sample in the first principal component (abscissa); PCA2 is the significance of the sample on the second principal component (ordinate). The *P* value represents the significance of the sample in this principal component. **b** Postoperative comparison of OTU level between the two groups. PCA1 is the significance of the sample in the first principal component (abscissa); PCA2 is the significance of the sample on the second principal component (ordinate)

Between Pre-F group and Pre-G group, flora composition indicated by OTU was not significantly different (Fig. 1a); between Post-F group and Post-G group, flora composition indicated by OTU was different in a certain degree (Fig. 1b). As visually shown, OTU was not significantly different between Pre-F group and Pre-G group; preliminarily indicating that intestinal flora was basically same between these two groups before the surgery. As shown by postoperative OTU diagram, interquartile range between primary and secondary main composition indicated by OTU was obviously widened in Post-G group; indicating that the diversity of flora in Post-G group was better than that in Post-F group.

### Alpha diversity analysis

Alpha diversity analysis is the diversity analysis of species in the sample. Generally, observed species index is adopted for such analysis, which indicates the actual level of OTU and reflects the diversity conditions of species in each sample. After the surgery, the structural diversity of flora was overall different between group G and group F. Rank sum test was performed on observed species index for Post-G group and Post-F group, which was statistically different (*P* < 0.05); observed species index in Post-G group was greater than that in Post-F group. Observed species index in group Pre-G was smaller than that in Pre-F subgroup, which was not statistically different. Therefore, after the surgery, in group G, the diversity of intestinal flora was better restored.

**Fig. 2:**
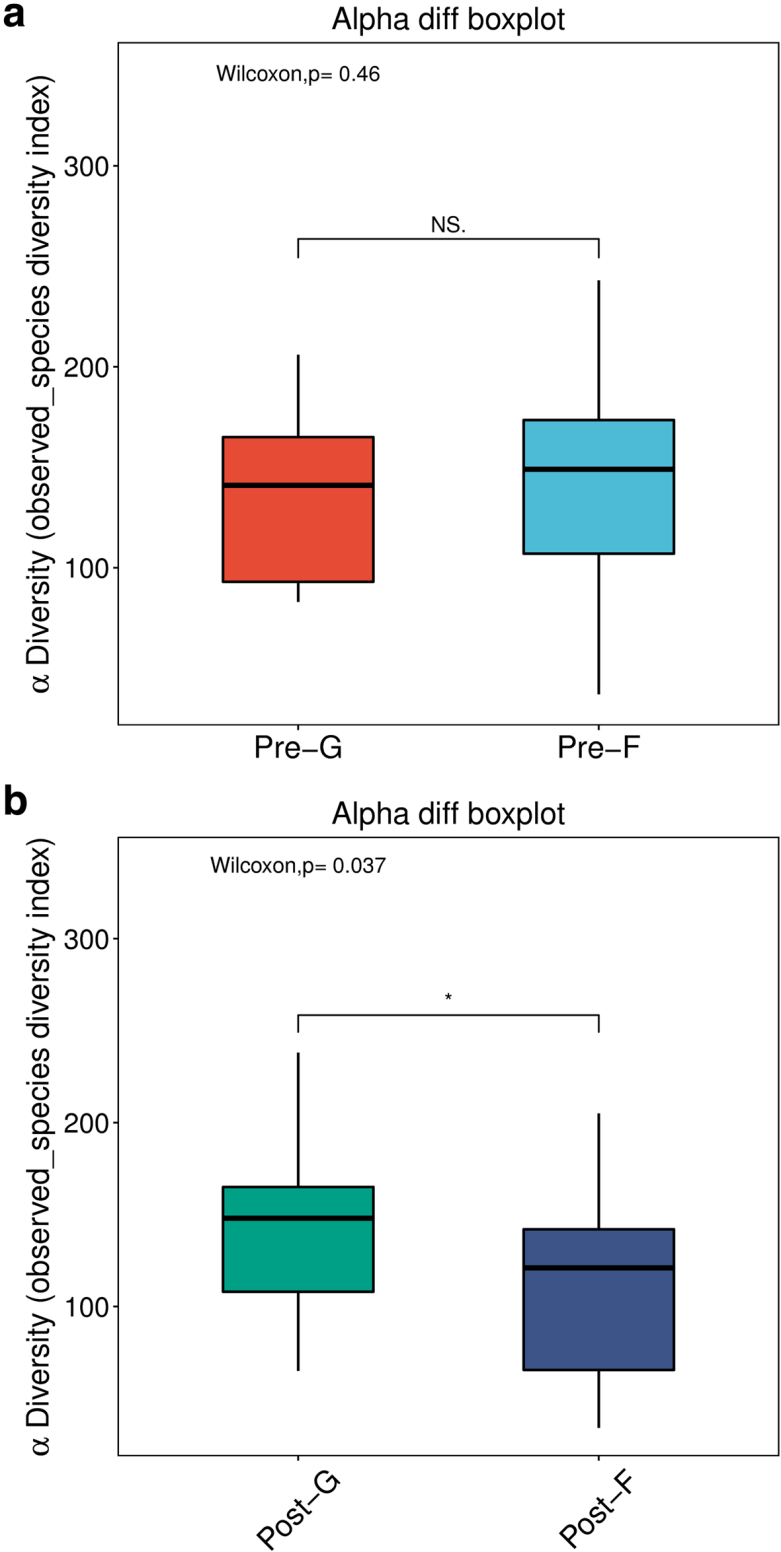
**a** Preoperative alpha diversity analysis. **b** Postoperative alpha diversity analysis

After the surgery, the structural diversity of flora was overall different between group G and group F (Fig. 2a&b).

### Beta diversity analysis

Through Beta diversity analysis, the difference value of each sample in the diversity of species was compared in quantitative or semi-quantitative way. In this study, Beta diversity analysis was made to further analyze the difference value of flora between two groups after the surgery. As shown by Alpha diversity analysis, the abundance and composition of intestinal flora were not statistically significant between two groups before the surgery. Therefore, Beta diversity analysis was performed only on the samples in Post-G group and Post-F group. In this study, Beta diversity analysis was performed through weighted Unifrac method and Adonis method.

### Weighted Unifrac method

**Fig. 3:**
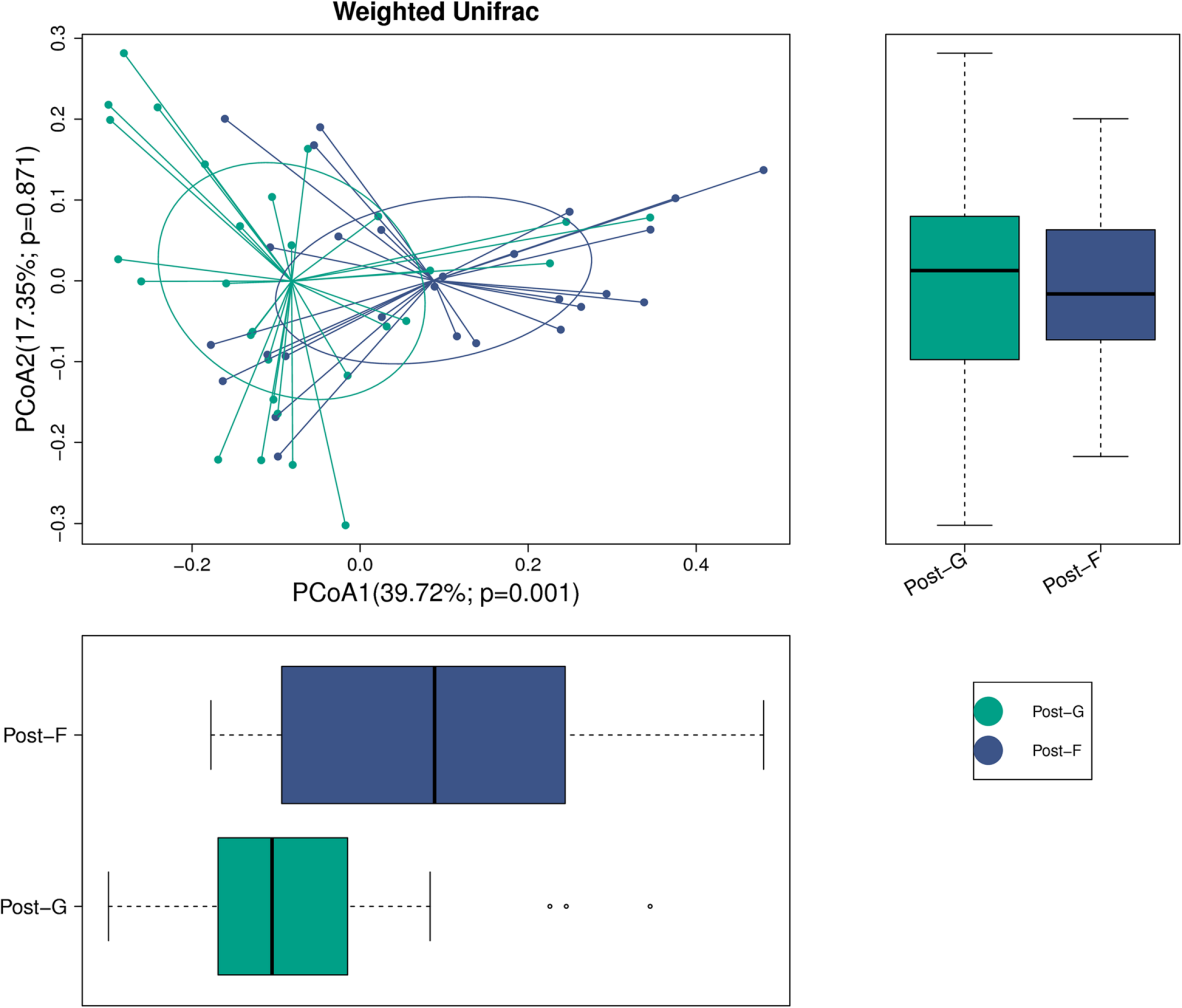
Weighted Unifrac diversity analysis of Post-G and Post-F beta

In the weighted Unifrac method, the difference value for species of flora between samples was compared through the information of system evolution [19]. By considering the abundance of sample sequence, a weighted processing was made (Fig. 3). Through principal coordinates analysis (PCoA), the difference value between samples was indicated.

Each point in the figure represents a sample, and the same circle represents the same group of samples. Out-circle points are discrete samples. The abscissa and ordinate represent the first and second principal coordinates, respectively; the percentage represents the contribution rate of the component of the coordinate to the sample difference; and the *p* value is the test p value of the corresponding principal coordinates. As shown in the figure, there was a significant difference in the first principal component between post-G and post-F (*P* = 0.001).

### Adonis analysis

**Fig. 4:**
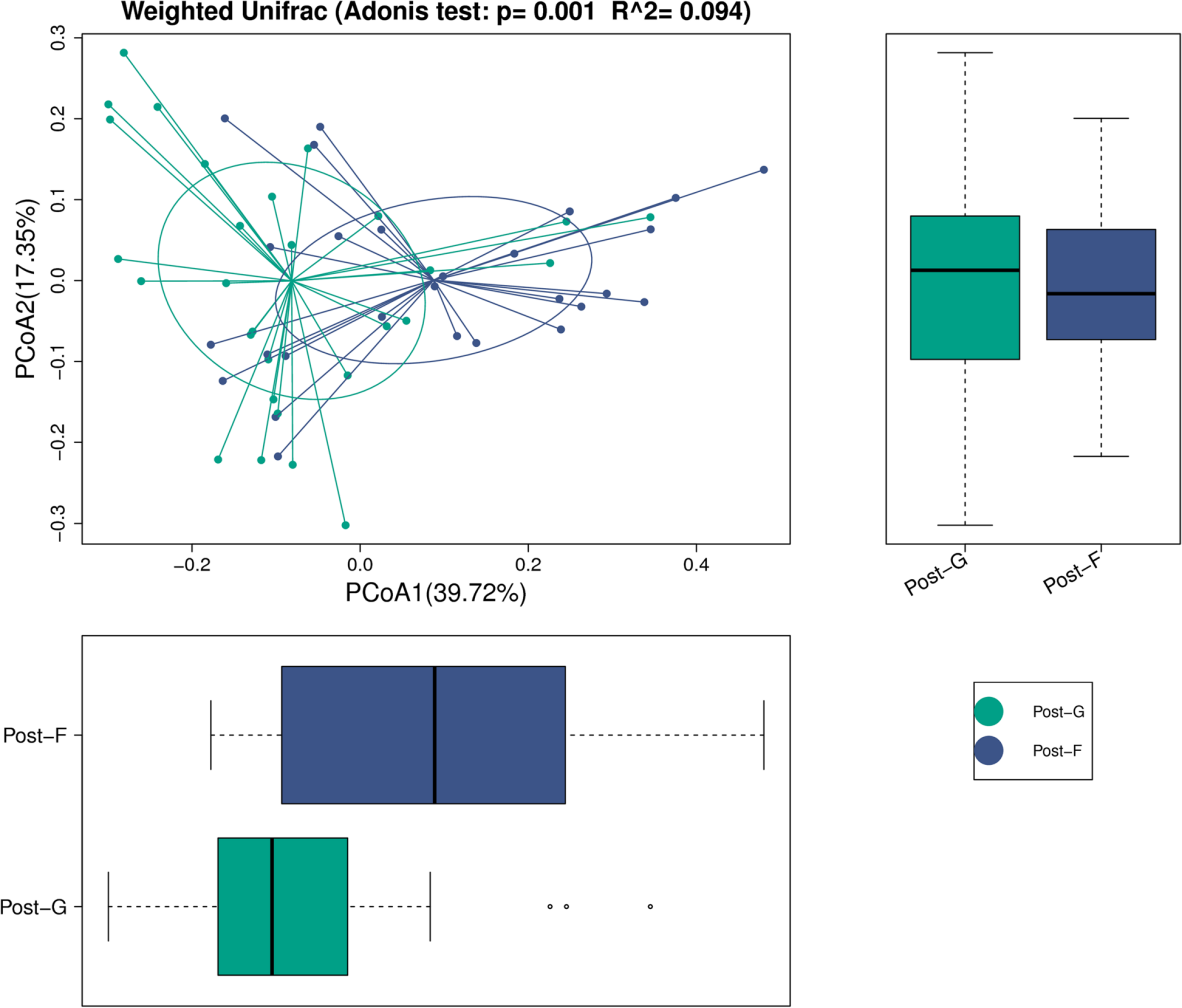
Adonis analysis

Weighted Unifrac method was an analytical method of too qualitative description. In this study, Adonis analysis was also performed. Through the linear model analysis, the dilution degree of different grouping factors for difference between samples was determined; through the permutation test, the significance was analyzed. R value indicated the dilution degree of different grouping factors for difference between samples, i.e. the ratio of variance to population variance in grouping factors; *P* value indicated the reliability of this analysis (Fig. 4). Through R Programming Language software, R value and P value were calculated.

As shown by the analysis through Adonis method, the diversity of intestinal flora was different between Post-G group and Post-F group.

### Analysis of species difference

**Fig. 5:**
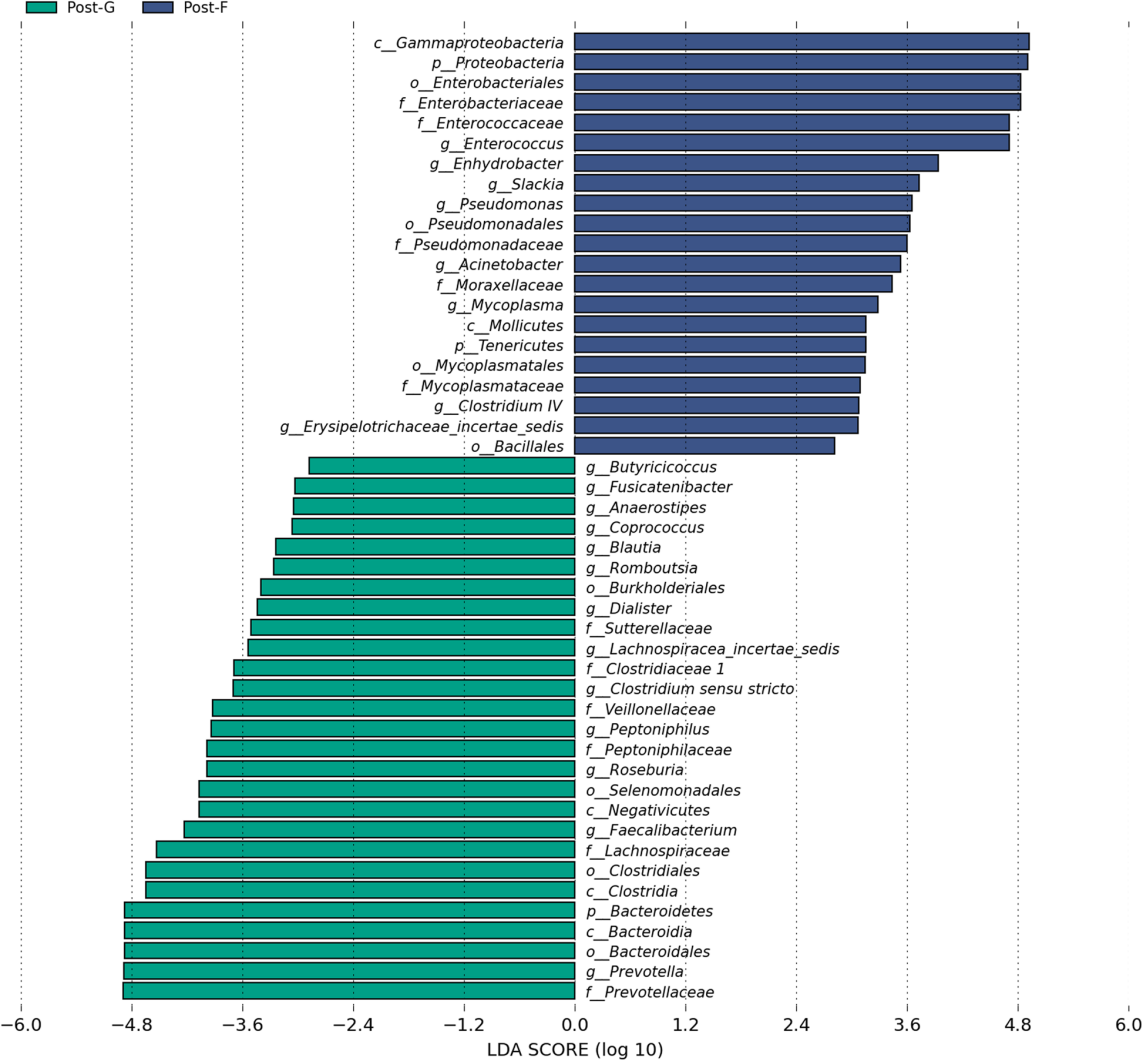
LDA graph

Only after core flora with a difference after the perioperative volumetric therapy of different regimens is found, study direction can be further determined. In this study, analytical method for species difference at the level of genus was adopted (Fig. 5).

LDA is the linear discriminant analysis diagram, which shows the differences between two groups of samples at the genus level, and the line length represents the significance of the differences.

**Fig. 6:**
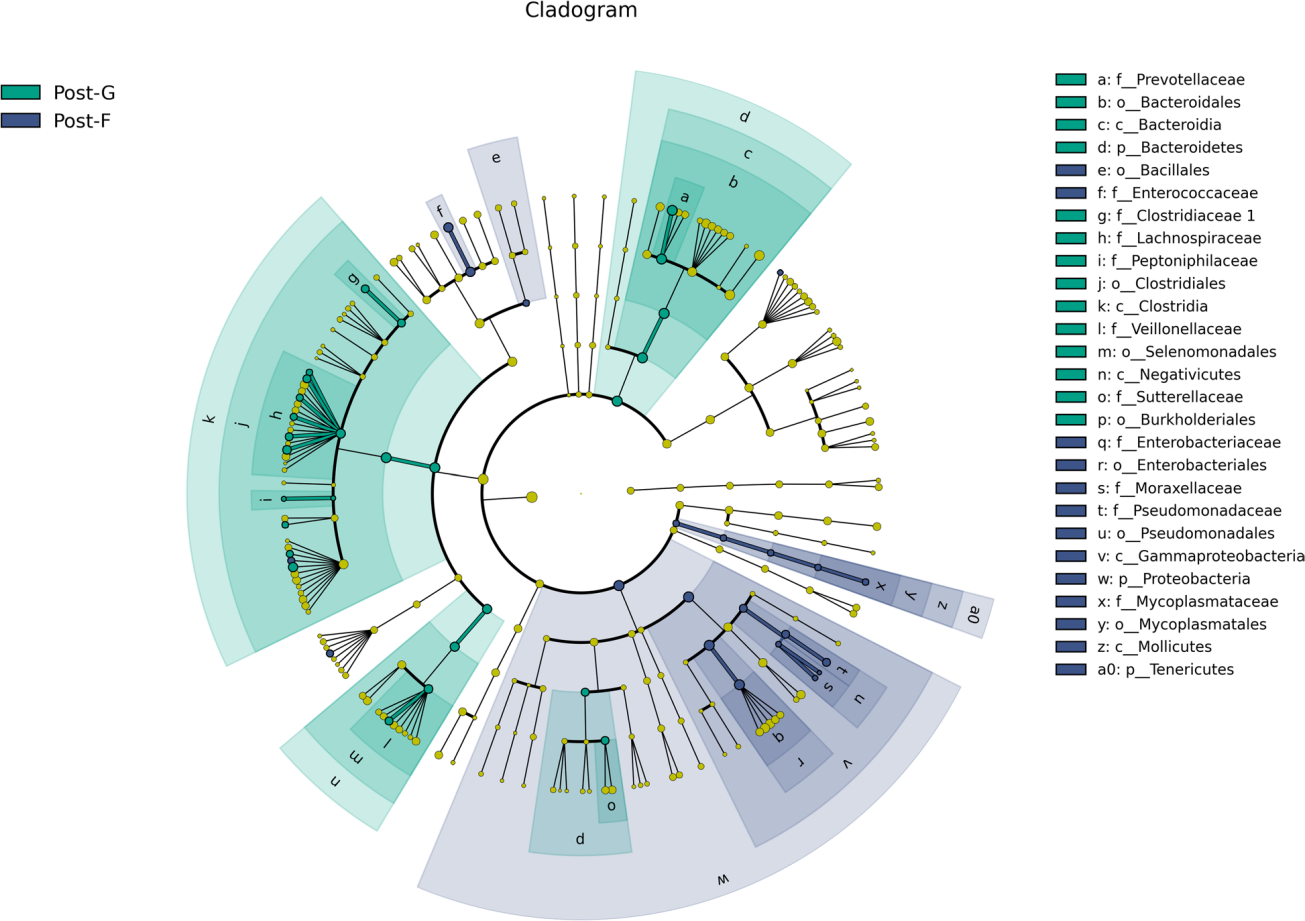
LEfSe graph

Through LDA effective size analysis (Fig. 6), two or more subgroups are compared; statistical significance and biological relevance are emphasized; biological marker of statistical difference between groups can be found. Blue region indicated Post_F group; green region indicated Post_G group. The node with same color as that of corresponding subgroup indicated the species of flora with important role in the corresponding group. Yellow node indicated the species of flora without important role in both groups.

Conditions of important bacteria causing a difference in flora between two groups. Bacteria with important role in causing a difference in flora in group G (*P* < 0.05) are: p*revotella, roseburia, lachnospiracea incertae sedis*. Bacteria with important role in causing a difference in flora in group F (*P* < 0.05) are: *enterococcus, pseudomonas aeruginosa, acinetobacter baumanni*i.

## Discussion

In the past, the perioperative target organs for fluid therapy were often focused on heart, lung, kidney, brain and less on intestinal function and intestinal flora. In this study, the conditions of intestinal flora were explored for the first time after the implementation of GDFT and LFI in the perioperative patients with pancreas tumor.

In perioperative period, intestinal mucosa barrier is easily impaired to cause a translocation of intestinal flora [20]. Subsequently, opportunistic pathogenic bacteria are multiplied; endotoxin is released; and finally, a series of pathophysiological course unfavorable for recovery of patients is caused. In this study, the restoration of postoperative intestinal flora in group G was superior to that in group F; the bacteria with important role in causing a difference were mainly some bacteria participating in anaerobic glycolysis and organic metabolism in group G, but were mostly opportunistic pathogenic bacteria in group F. Therefore, perioperative benefit in group G might be greater than that in group F possibly for the following reasons: since fluid infusion was made at more uniform velocity in GDFT group, the insufficient perfusion of microcirculation was more difficult to occur in intestinal cavity during the long-time surgery [21]; in some patients in GDFT group, Dopamine was given at small dose during the surgery, which dilated the visceral vessel to better supply blood/oxygen for intestinal tissue during the longer-time surgery [22, 23]. However, as also shown by some studies [24–26], visceral vessel was not obviously influenced after the administration of Dopamine. Therefore, more precise method should be obtained to assess the conditions of blood supply in mesenteric vessel.

As shown by recent studies [27], long-term survival rate of patients with pancreas tumor was closely related to intestinal flora. In the patients of pancreas tumor with a long survival period, intestinal flora is of higher alpha diversity. Therefore, by maintaining the diversity of intestinal flora in perioperative patients, the prognosis of patients will certainly be benefited. According to the characteristics of intestinal flora, long-term survival can be predicted in the patients with pancreas tumor, particularly, with the existence of *alpha proobacteria, sphingoo bacteria* and *flavobacteria,* the long-term survival is benefited after the surgery on pancreas tumor. In this study, such flora was not significantly different between two groups; in GDFT group, alpha diversity of intestinal flora was more protected. However, the following aspects are worthy of further study: whether the long-term survival of patients with pancreas tumor is also benefited after the restoration of such diversity; whether the long-term survival of other specific populations is benefited by other bacteria, and whether the postoperative length of stay will get affected with the change in flora in the short-term.

In group G, *prevotella* was one of bacteria with important role in causing a difference in flora. *Prevotella* participates in the metabolism; it also secretes *trimetlylamine oxide* (TMAO), which potentially influences the activity of platelet and the thrombosis. In other words, there is an intrinsic relation among intestinal flora, TMAO and thrombosis [28]. *Prevotella* also facilitates the restoration of coagulation function after the surgery [29, 30]. As also shown by some studies [31–33], after the supplementation of probiotics for a short time, TMAO level in human body was less influenced; and coagulation function was not improved. Therefore, from the angle of intestinal flora, it is still worthy of exploration whether the restoration of coagulation function is influenced after the implementation of different regimens of perioperative volumetric therapy. *Roseburia* participates in the glycolysis of various carbohydrates. Its catabolic products mainly include: butyric acid and butyrate. *Lachnospiracea incertae sedis* participates in the glycolysis of organic nutrients and sugar in human body. In group F, *enterococcus, pseudomonas aeruginosa and acinetobacter baumanni*i are with important role in causing difference in flora. They are opportunistic pathogenic bacteria.

Intestinal flora is influenced in a certain degree by the secretion of pancreatic juice. In this study, although pancreatin enteric-coated capsules (Creon) was given daily, its inhibitory effect for secretion of pancreatic juice could not be assessed accurately.

## Conclusion

In patients undergoing pancreatic tumor surgeries, the protection of intestinal flora and promotion of postoperative recovery can be achieved by implementation of GDFT and LFI. Through GDFT, the diversity of intestinal flora is better restored after the surgery; through GDFT and LFI, dominant intestinal flora is different after the surgery.

## Data Availability

The datasets generated and/or analysed during the current study are available in the national center for biotechnology information (NCBI) repository, BioProject ID PRJNA823673.

## Abbreviations

LFI: Liberal fluid infusion
GDFT: Goal-directed fluid therapy
SVV: Stroke volume variation
CI: Cardiac index
OTU: Operational taxonomic unit
TMAO: Trimetlylamine oxide
